# From Microbiota to Cancer: Role of Extracellular Vesicles in Gut–Lung Axis

**DOI:** 10.3390/cancers17243946

**Published:** 2025-12-10

**Authors:** Giusy Daniela Albano, Simona Taverna

**Affiliations:** Institute of Translational Pharmacology (IFT), National Research Council (CNR) of Italy, 90146 Palermo, Italy

**Keywords:** extracellular vesicles, microbiota, gut–lung axis, non-small cell lung cancer

## Abstract

Lung cancer (LC) remains the leading cause of cancer-related mortality worldwide, and resistance to therapy continues to pose a major clinical challenge. Increasing evidence highlights the relevance of the gut–lung axis in immune response modulation, tumor progression, and treatment outcomes. Within this inter-organ network, bacterial extracellular vesicles (bEVs), nanosized particles containing proteins, nucleic acids, and metabolites, serve as important mediators of host–microbiota communication, influencing immune regulation, metabolic pathways, and tumor biology. This review explores EV-mediated mechanisms involved in LC pathogenesis, including immune modulation, epigenetic regulation, and microbial metabolite signaling. The mechanistic influence of environmental and dietary factors on bEV composition and function is further explored, and emerging translational applications, ranging from diagnostic biomarker development to drug delivery strategies and modulation of immunotherapy responses, are discussed. Moreover, ongoing clinical trials testing microbiota-based strategies in non-small cell lung cancer (NSCLC) are summarized, offering potential new perspectives for personalized cancer management.

## Simple Summary

Lung cancer is the leading cause of cancer-related deaths worldwide, and many patients develop resistance to treatment. Recent studies show that microorganisms living in the gut and lung can influence inflammation, tumor growth, and how patients respond to therapy. Bacteria release extracellular vesicles (bEVs), nanoparticles that can carry signals affecting both immune cells and cancer cells. This review explores how microbial vesicles may influence lung cancer development and progression and highlights their promising role as biomarkers and potential new therapeutic tools.

## 1. Introduction

In recent years, the discovery of a distinct and dynamic microbial community within the lungs has fundamentally transformed the understanding of respiratory health and disease. Once considered sterile, currently lungs are known to harbor a low-biomass but immunologically active microbiota that interacts with host pathways, modulates inflammatory responses, and potentially contributes to tumor biology [1,2]. In parallel, the gastrointestinal (GI) tract, which hosts the most various and densely populated microbiota in the human body, serves as a critical regulator of systemic immunity. Bidirectional communication between the gut and lung mucosa, termed the gut–lung axis, plays a pivotal role in modulating immune responses and cancer biology [3,4]. Microbial metabolites, immune cell trafficking, and epigenetic regulators disseminate signals systemically, influencing the lung tumor microenvironment (TME) and therapeutic responses. Lung cancer (LC), predominantly non-small cell lung cancer (NSCLC), remains the leading cause of cancer-related mortality worldwide. Although treatments such as immune checkpoint inhibitors (ICIs) have improved outcomes, resistance and variable clinical responses persist, highlighting the need to better understand how the gut–lung axis affects tumor immunity and progression. Among the mediators of inter-organ communication, extracellular vesicles (EVs) secreted by host and microbial cells have emerged as key players, carrying bioactive cargos that regulate immune modulation and tumor progression [5,6]. EVs are membrane-bound nanoparticles released by all cell types, and they are essential for intercellular communication [7]. Recent guidelines from the International Society for Extracellular Vesicles (ISEV) have standardized their isolation and characterization, supporting clinical translation [8]. Host-derived EVs hold promise for early diagnosis, disease monitoring, and revealing tumor heterogeneity, drug resistance, and disease progression [9,10]. The bEVs released from prokaryotic membranes carry unique cargos such as lipopolysaccharides (LPSs), peptidoglycans, bacterial metabolites, and virulence factors. These components enable bEVs to interact with host epithelial and immune cells through pathways distinct from those activated by host EVs, often eliciting strong pro-inflammatory responses, modulating innate immunity, and influencing oncogenic signaling [11]. bEVs can disseminate systemically and reach distal organs, including the lungs, independently of viable bacteria, acting as potent mediators of microbe–host communication [12]. These mechanistic specificities provide a compelling rationale for examining bEVs as contributors to lung carcinogenesis. Recent evidence indicates that bEVs are drivers of cancer progression through multiple complementary mechanisms. bEVs actively remodel the TME by promoting immunosuppression, macrophage M2 polarization, and inhibition of dendritic cell maturation, thereby facilitating tumor immune evasion. They also deliver genetic and epigenetic regulators including DNA, microRNAs (miRNAs), virulence factors, and chromatin-modifying enzymes that modulate oncogene activation, suppress tumor suppressor pathways, and modulate host transcriptional programs. In addition, bEVs promote angiogenesis and metastasis by inducing VEGF expression, transporting matrix metalloproteinases, and triggering the epithelial–mesenchymal transition (EMT). Their cargo, which is enriched in pathogen-associated molecular patterns (PAMPs), activates nuclear factor kappa-light-chain-enhancer of activated B cells (NF-κB) and Mitogen-Activated Protein Kinase (MAPK) signaling pathways, sustaining chronic inflammation, oxidative stress, and DNA damage, all of which contribute to tumor initiation and progression [13,14].

**Figure 1:**
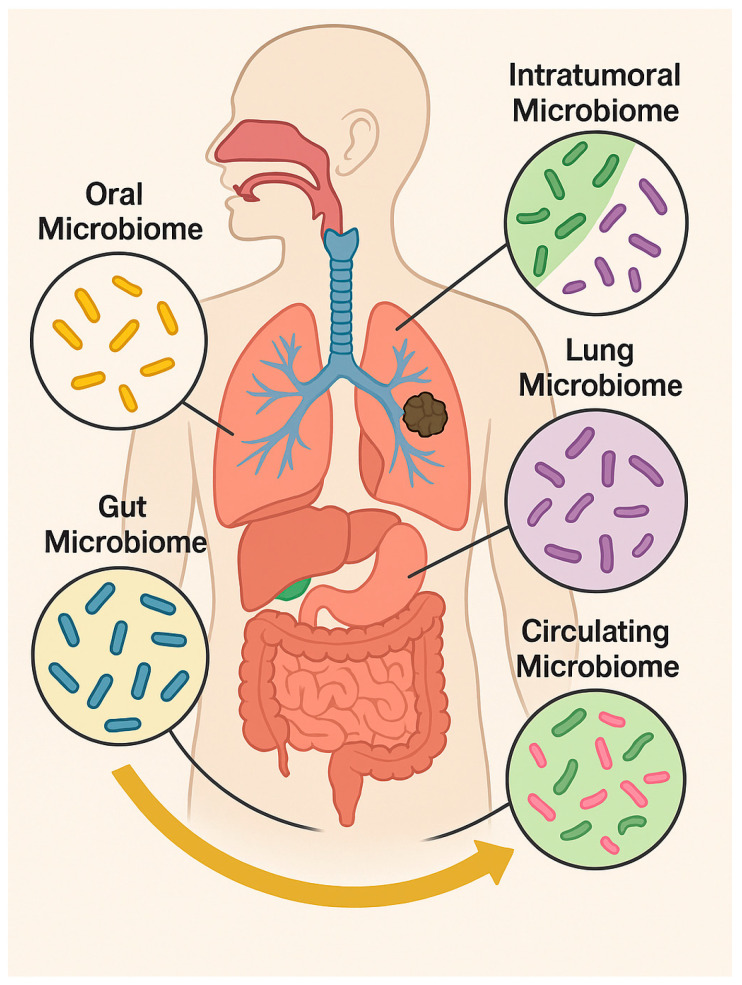
Distribution of the main human microbiomes across body compartments (oral, gut, pulmonary, circulating, and intratumoral) and their interactions influencing health, immunity, and tumor progression. Microsoft Copilot was used for the graphical rendering of the figure.

Emerging evidence further highlights that distinct microbial niches, including the oral cavity, gut, lungs, and intratumoral and circulating microbiome, contribute to LC pathogenesis and therapy responses [15]. The oral microbiome serves as a microbial reservoir, seeding the lower airways and influencing local inflammation [16]. Dysbiosis, characterized by the overgrowth of pathogens such as *Fusobacterium nucleatum*, *Porphyromonas gingivalis*, and *Prevotella* species, promotes chronic inflammation and facilitates the translocation of bacteria or their metabolites to the lower respiratory tract via micro-aspiration. Once in the lung microenvironment, these microbes modulate epithelial signaling, enhance immune evasion, and activate tumor-promoting pathways such as NF-κB, signal transducer and activator of transcription 3 (STAT3), and β-catenin [16]. Bacterial virulence factors contribute to DNA damage, impaired apoptosis, and remodeling of the TME, creating conditions favorable for malignant transformation [17]. The gut microbiota modulates systemic immunity and controls antitumor responses, particularly in the context of immune checkpoint blockade. The lung microbiome directly affects epithelial homeostasis and the immune tone, while intratumoral microbes influence the TME by modulating cytokine production, angiogenesis, and immune cell infiltration [3]. Additionally, circulating microbial DNA and EVs reflect microbial translocation and may serve as non-invasive biomarkers or mediators of distant immune modulation [18] (Figure 1).

This review provides an updated and integrative analysis of how gut- and lung-derived microbial communities contribute to LC initiation and progression, with a specific emphasis on the emerging role of EV-mediated signaling. Unlike previous reviews on the gut–lung axis or microbiome–cancer interactions, this work offers a “vesicle-centred” perspective by focusing specifically on microbiota-derived EVs and their mechanistic influence on lung carcinogenesis. By integrating immune, metabolic, and epigenetic pathways activated by microbial vesicles and examining how environmental and dietary exposures remodel bEV cargo and function, this review offers a distinct and translationally relevant framework for understanding EV-mediated microbiota–host interactions in NSCLC prevention, diagnosis, and therapy.

## 2. Lung Cancer and the Gastrointestinal Microbiota: The Gut–Lung Axis

**Figure 2:**
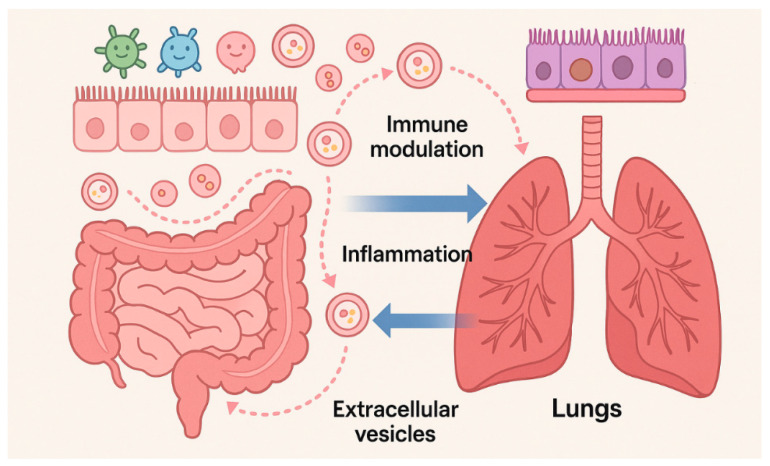
Bidirectional gut–lung communication mediated by microbiota-derived EVs. Gut and lung EVs transport microbial metabolites, nucleic acids, and immune modulators, altering epithelial barriers, immune responses, and microbial composition, thereby linking inter-organ crosstalk to inflammation and tumorigenesis. Microsoft Copilot was used for the graphical rendering of the figure.

The gut–lung axis constitutes a bidirectional network of metabolic, microbial, and immune interactions. [19]. Increasing evidence indicates that this inter-organ communication critically shapes NSCLC onset, progression, and therapeutic responsiveness [3,4]. Central to this crosstalk are short-chain fatty acids (SCFAs), which are produced by anaerobic fermentation of dietary fibers by commensal gut bacteria. SCFAs modulate pulmonary immunity by inhibiting histone deacetylases, influencing gene expression, promoting dendritic cell maturation, and enhancing CD8^+^ T-cell cytotoxic activity, thereby supporting antitumor responses [20,21,22]. In parallel, microbe-associated molecular patterns (MAMPs) engage pattern recognition receptors (PRRs) such as Toll-like receptors (TLRs) and Nucleotide Oligomerization Domain (NOD)-like receptors in the lungs to regulate cytokine production, immune cell recruitment, and polarization within the TME [4,23]. Diet is a major determinant of microbiota composition and LC outcomes. Adherence to a Mediterranean diet rich in plant fibers, polyphenols, and unsaturated fats promotes microbial diversity and expansion of beneficial taxa such as *Akkermansia muciniphila*, *Bacteroides*, and *Faecalibacterium prausnitzii*, which correlate with improved immune surveillance and enhanced responses to immune checkpoint inhibitors [3,24]. *Akkermansia muciniphila*, normally comprising 1–4% of healthy gut microbiota, ferments inulin and other fibers into SCFAs that reinforce epithelial barrier integrity and modulate T-cell responses [25]. Preclinical models confirm that high-fiber diets enrich *Akkermansia muciniphila* and *Bifidobacterium pseudolongum*, reduce immunosuppressive cell populations, and attenuate lung tumor growth [26]. Consistently, clinical studies in NSCLC patients show that elevated *Akkermansia* levels are associate with favorable RECIST outcomes in NSCLC patients receiving immunotherapy [27,28,29]. Plant-forward dietary patterns such as the Planetary Health Diet are similarly linked to reduced LC incidence and mortality, mediated by microbiota-derived metabolites, including SCFAs, bile acid derivatives, and tryptophan catabolites, that exert systemic immunomodulatory effects [30]. Conversely, gut dysbiosis characterized by reduced α-diversity and an expansion of pathogenic taxa such as *Clostridium* spp. is associated with immunotherapy resistance and poor clinical outcomes [3,21]. Dysbiosis compromises epithelial barrier integrity and facilitates the translocation of bacterial components such as LPSs, which activate Pattern Recognition Receptor (PRR)-dependent NF-κB and STAT3 signaling, thereby promoting chronic inflammation, tumor survival, angiogenesis, and metastasis [31,32]. Loss of SCFA-producing bacteria also impairs regulatory T-cell differentiation and mucosal defense, forming a tumor-promoting microenvironment [20]. Dietary and environmental exposures not only modulate microbial composition but also influence the bEV cargo. High-fiber diets enrich SCFA-producing taxa, resulting in bEVs enriched with butyrate and propionate that promote regulatory T-cell differentiation and dampen inflammation [33]. In contrast, cigarette smoke profoundly alters the epithelial exosome composition; EVs from smoke-exposed airway epithelial cells transfer the lncRNA MEG3 and induce macrophage pyroptosis through the TREM-1/METTL3 axis, exacerbating lung injury [34]. Antibiotic exposure reduces microbial diversity and bEV production, potentially impairing immune priming and decreasing ICI responsiveness [35]. These findings highlight the need to consider diet, environmental pollutants, and medication use when interpreting vesicle-based biomarkers or designing microbiota-targeted interventions. Growing evidence indicates that bEVs are key effectors of the gut–lung axis. Gut-derived EVs can cross the intestinal barrier, enter systemic circulation, and deliver microbial proteins, LPS, and nucleic acids to distant organs including the lungs [6]. Within the pulmonary microenvironment, these vesicles interact with alveolar macrophages and dendritic cells and modulate cytokine release via TLR signaling, as well as both inflammatory and antitumor responses. This vesicle-mediated communication complement established mediators such as SCFAs and provides a mechanistic rationale for how the gut microbiota exert direct influence on lung immunity and potentially induce lung carcinogenesis [36]. Clinical and translational findings support a role for SCFAs in LC biology. A prospective study in LC patients receiving chemotherapy or targeted therapy reported higher serum SCFA levels, particularly acetate and isobutyrate, in treatment responders. Mechanistic experiments using the A549 cell line revealed that isobutyrate inhibits proliferation, migration, and invasion; induces apoptosis and cell-cycle arrest; and modulates GPR41/43 and histone acetylation [37]. Multiple independent studies consistently show depletion of SCFA-producing taxa in NSCLC patients, indirectly suggesting reduced SCFA availability [38]. Additional data suggest that microbiome-derived metabolites, including SCFAs, may influence long-term therapeutic efficacy and late adverse events in LC survivors [39]. Environmental stressors such as smoking, air pollution, and a high-fat/low-fiber diet exacerbate dysbiosis and systemic inflammation, thereby increasing LC susceptibility [21,23]. Within this intricate network, EVs act as nanoscale mediators of gut–lung communication by transporting microbial metabolites, nucleic acids, and immune modulators that influence epithelial permeability, T-cell priming, and cytokine networks [6,10,40]. EVs therefore represent an underexplored but potentially crucial link in gut–lung–cancer interactions (Figure 2).

## 3. Risk Factors: Pollution, Lifestyle, and Inflammation

Environmental and lifestyle factors critically increase LC risk by disrupting epithelial barrier integrity and perturbing the gut–lung microbial axis, thereby influencing chronic inflammation and carcinogenesis [41,42,43].

Airborne and ingested pollutants such as particulate matter (PM2.5 and PM10), nitrogen oxides (NOₓ), ozone (O_3_), combustion-derived carbon particles, and micro/nano-plastics cause structural and functional damage to respiratory and GI epithelia. These pollutants impair tight junction proteins such as claudins, occludin, and ZO-1; compromise mucociliary clearance; and increase epithelial permeability, facilitating pathogen retention and accumulation of pro-inflammatory stimulus in airway surface liquids [44,45,46,47,48]. Consequently, impaired mucociliary function leads to mucus hypersecretion, microbial overgrowth, and sustained airway inflammation [46,49,50]. PM2.5 induces oxidative stress, as well as mitochondrial and DNA damage, by activating key signaling pathways involving interleukin (IL)-6, IL-1β, tumor necrosis factor-alpha (TNF-α), and chemokines such as IL-8 and chemokine (C-X-C motif) ligand 1 (CXCL1) and by recruiting immune cells including neutrophils and macrophages [46].

Furthermore, PM modulates airway epithelial plasticity, influencing the EMT and the mesenchymal–epithelial transition (MET). Acute PM exposure activates the aryl hydrocarbon receptor (AhR) and STAT3, suppresses Notch1, and favors ciliated epithelial differentiation [51,52], whereas chronic exposure engages the notch1/2/3, Wnt/β-catenin, and Transforming Growth Factor (TGF)-β pathways, promoting the EMT and preneoplastic remodeling [46].

While direct comparisons across NSCLC histological subtypes remain limited, indirect evidence indicates that adenocarcinoma may be particularly susceptible. Arising from peripheral alveolar and bronchiolar epithelium, adenocarcinoma develops in regions where epithelial cells are especially vulnerable to PM-induced disruption of tight and adherent junctions [53]. Epidemiological data associate PM2.5 and NO_2_ exposure more strongly with adenocarcinoma than with squamous cell carcinoma, and in vitro studies demonstrate heightened barrier perturbation in type II alveolar epithelial cells [54,55]. These observations suggest a potential subtype-specific effect of environmental pollutants on epithelial integrity, underscoring the need for direct comparative analyses. Pollution also perturbs the lung and gut microbiota, reducing microbial diversity and SCFA-producing taxa while enriching pro-inflammatory microbial populations.

This dysbiosis decreases butyrate and other beneficial metabolites, impairing G protein-coupled receptor (GPCR)-mediated anti-inflammatory signaling and Histone Deacetylase (HDAC) activity, thereby enhancing pro-inflammatory gene expression and epithelial damage [44,56,57]. The gut–lung axis facilitates systemic dissemination of microbial products and cytokines, modulating the lung immune tone and the TME [58]. Co-exposure to a Western diet (low fiber, high fat) and air pollution exacerbates dysbiosis, endotoxemia, and inflammatory cascades, further reducing SCFA levels and amplifying systemic inflammation [4,59]. Dietary patterns also influence bEVs, affecting host susceptibility to diseases [60,61].

The pollution-induced dysbiosis alters the cargo and release of microbial and host EVs, which transport pro-inflammatory miRNAs, cytokines, and oxidative signals from the gut to the lung. This mechanisms integrates environmental insults into systemic immune activation and epithelial stress responses, contributing to gut–lung-axis-driven tumorigenesis [6,10]. Overall, these findings highlight the complex interplay among pollutants, diet, and microbiota in orchestrating chronic inflammation, immune dysregulation, and neoplastic transformation.

## 4. Extracellular Vesicles in Lung Cancer: Host and Bacterial Contributions

EVs contribute to LC progression by facilitating communication between tumor cells, host tissues, and gut–lung bacteria. Tumor-derived EVs (tEVs) promote immune evasion and therapy resistance, while bEVs can modulate inflammation and influence tumor behavior. Together, these host and microbial signals create an interconnected network that drives disease and may provide useful biomarkers or therapeutic targets.

### 4.1. Tumor-Derived EVs (tEVs) and Their Roles in Lung Cancer Progression

EVs are nanosized, membrane-bound particles that deliver signals to epithelial, immune, and tumor cells. In LC, tumor-derived EVs (tEVs) and bEVs remodel the TME, induce systemic immunity, and facilitate bidirectional communication along the gut–lung axis [8,62]. The tEVs constitute a heterogeneous population of nanoscale vesicles carrying proteins, lipids, DNA, and non-coding RNAs, including numerous oncogenic and immunomodulatory miRNAs [8,62,63].

Through the transfer of oncogenic proteins, metabolites, DNA fragments, and regulatory RNAs, tEVs modulate immune evasion, angiogenesis, stromal remodeling, and pre-metastatic niche formation. Their biogenesis and cargo loading are dynamically regulated by the TME and therapeutic pressure, and their stability in biofluids supports their use as minimally invasive biomarkers for early detection and treatment monitoring [9,64]. Beyond local effects, tEVs engage in systemic crosstalk with the gut. Circulating tEVs reach the GI tract and influence epithelial homeostasis and mucosal immunity [65,66]. tEV-associated miRNAs and cytokines downregulate tight junction proteins, increase intestinal permeability, and alter the microbiota’s composition and metabolism [67]. This tEV-driven dysbiosis increases the systemic translocation of bEVs, reinforcing a tumor-gut feedback loop that fuels lung tumor progression via the gut–lung axis [68]. tEVs profoundly remodel the systemic immune tone by transporting Programmed death-ligand 1 (PD-L1), TGF-β, IL-10, and immunosuppressive miRNAs that suppress T-cell activation, expand Tregs, and polarize macrophages toward an M2 phenotype [69,70]. These shifts modify the host’s responsiveness to gut- or airway-derived bEVs, altering susceptibility to bEV-induced inflammation or immunosuppression [71]. In pulmonary tissues, tEVs sensitize epithelial and immune cells to microbial signals by upregulating TLR4 and NF-κB signaling in response to LPS-containing bEVs, suppressing dendritic cell maturation, and altering macrophage polarization [42]. Evidence also supports reciprocal regulation: LPS-rich bEVs reprogram tumor cells by activating hypoxic and inflammatory pathways that alter tEV secretion and cargo composition [72]. This feed-forward loop contributes to interindividual variability in responses to ICIs and chemotherapy, partly through modulation of systemic immunity and the microbiota composition [73]. tEV-associated PD-L1 may counteract the immunostimulatory effects of favorable gut microbiota linked to enhanced ICI efficacy [74]. Elevated circulating EV-associated PD-L1 levels correlate with a poor prognosis and a reduced ICI response in NSCLC, although standardized clinical thresholds remain to be defined [69]. While bEVs have been implicated in promoting chemotherapy resistance, quantitative mechanistic data on NSCLC remain limited. In vitro, bEV exposure can increase cisplatin’s IC_50_ 1.5–2-fold and upregulate Bcl-2, supporting a role in drug resistance [75].

### 4.2. Bacterial Extracellular Vesicles: Origin, Composition, and Impact on Lung Cancer

Microbiota-derived EVs include Gram-negative (OMVs) and Gram-positive MVs, collectively referred to as bEVs [76,77].

Emerging analytical technologies, including lipidomics exploiting bacterial lipid signatures (LPS, lipid A, and branched-chain fatty acids) [78], acoustic nano-filtering [79], and high-resolution/nano-flow cytometry [80], enable discrimination of bEVs from host EVs and improve vesicle characterization. The bEVs are able to cross epithelial barriers, allowing systemic dissemination to distal organs, including the lungs [5,81]. In healthy individuals, bEVs mediate interbacterial communication and horizontal gene transfer, but dysbiosis enhances their pro-inflammatory and tumor-promoting effects. LPS-rich bEVs activate TLR4/NF-κB signaling, driving cytokine production and chronic inflammation associated with tumor initiation [82]. bEVs can cross the gut barrier via paracellular and transcellular pathways [83,84], enter circulation either directly or through immune cell transport [85], and modulate lung immunity [86]. In pulmonary tissues, gut-derived or inhaled bEVs accumulate and modulate airway immunity. Indoor dust is a reservoir of bEVs, and elevated serum antibodies against dust-associated bEVs correlate with asthma, COPD, and LC [87,88,89,90]. Gram-negative bEVs from *Pseudomonas aeruginosa* and *Escherichia coli* preferentially induce Th17 responses and neutrophilic inflammation, whereas Gram-positive *Staphylococcus aureus* bEVs elicit Th1-skewed inflammation linked to fibrosis and emphysema [91,92,93].

#### 4.2.1. Molecular Mechanisms of bEV-Mediated Tumor Promotion and Therapy Resistance

The bEVs promote tumorigenesis by inducing oxidative stress and DNA damage [14]; activating the PI3K/AKT, MAPK/ERK, and STAT3 pathways that support proliferation, EMT, and survival [94,95]; and remodeling the ECM via MMP delivery [13]. They modulate immune landscapes by recruiting Myeloid-derived suppressor cells (MDSCs) and M2 macrophages and inhibiting CD8^+^ T-cell cytotoxicity [96]. PRR signaling critically alters these effects. LPS-containing vesicles activate TLR4–NF-κB pathways and upregulate PD-L1 [89]. Gram-positive lipoprotein-rich bEVs signal through TLR2-MyD88 to promote Th1 responses [97]. *Bifidobacterium*-derived bEVs enhance PD-L1 expression in tumor cells and synergize with anti-programmed cell death protein 1 (PD-1) therapy in vivo. bEVs also regulate dendritic cell maturation and T-helper cell polarization, shaping Th1/Th17 versus Treg responses. Their metabolite cargo includes SCFAs, indoles, bile acids, and polyamines and signals through GPCRs and AhR to influence epithelial integrity and immune regulation [35]. bEV-associated sRNAs, such as those from Lactobacillus murinus, modulate host polyamine biosynthesis by targeting key metabolic enzymes [98]. The microbiota composition significantly influences ICI efficacy. The abundance of *Akkermansia muciniphila* or *Bifidobacterium* spp. correlates with improved response rates and survival in NSCLC [33,99,100], suggesting that bEV-mediated immunomodulation contributes to therapeutic outcomes. Although many bEVs exert pro-tumorigenic effects, others display antitumor properties. Certain bEVs promote M1 macrophage polarization, enhance dendritic cell maturation, and stimulate CTL activation [14,101]. Probiotic-derived EVs can induce ROS-mediated apoptosis and ER stress in tumor cells [102], and *Akkermansia muciniphila* bEVs drive histone acetylation and HSP70 expression while recruiting M1-like macrophages [103]. Thus, bEV effects are highly strain- and cargo-dependent.

#### 4.2.2. miRNAs and the Lung–Gut Microbiota Axis in Lung Cancer

The reciprocal regulation between host miRNAs and the microbiota significantly influences NSCLC pathogenesis [104]. Microbial metabolites, including SCFAs and LPS, modulate miRNA expression [105] and promote oncogenic miRNAs such as miR-21 and miR-155, key drivers of immune evasion, chemoresistance, and a poor prognosis [14,106], while dysbiosis suppresses tumor-suppressive miRNAs such as let-7 [107]. An altered lung microbiota, for instance the presence of *Streptococcus*, *Prevotella*, and *Veillonella*, affects the PI3K, MAPK, and ERK pathways through miRNAs, including miR-126, miR-133b, and miR-145 [108]. miR-133b also forms part of a feedback loop regulating microbial dysbiosis via TLR signaling [109]. bEVs selectively package miRNAs and deliver them to host cells, enabling microbiota-driven epigenetic reprogramming [63,110]. Examples include miR-21 transfer by *Fusobacterium nucleatum* in colorectal cancer [14], miR-155 delivery by *Helicobacter pylori* bEVs [111], and miR-21/miR-155-mediated oncogenic signaling in breast cancer [106,112]. Similar mechanisms likely contribute to miRNA dysregulation in NSCLC, positioning bEV-borne miRNAs as key modulators of the gut–lung axis (Table 1).

## 5. Biomedical Potential of bEVs in Lung Cancer Management

bEVs offer versatile biomedical applications in LC, spanning immunotherapy, targeted drug delivery, diagnostics, and nanovaccine development. Preclinical studies first demonstrated that bEVs can selectively accumulate in tumor tissues and trigger potent antitumor immune responses via interferon-gamma (IFN-γ) signaling in murine models.

Notably, IFN-γ exerts a dual role, as it can also induce immunosuppressive factors including immune checkpoint molecules within the TME, highlighting the context-dependent immunomodulatory potential of bEVs [114]. Gut microbiota-derived bEVs further modulate LC immunotherapy outcomes. Vesicles from commensal *Bifidobacterium* species (Bif-bEVs) are internalized by LC cells through dynamin-dependent endocytosis, upregulating PD-L1 via TLR4-NF-κB signaling and influencing the efficacy of anti-PD-1 immune checkpoint inhibitors [89]. Bif-bEVs effectively penetrate both murine intestinal organoids and patient-derived LC organoids, underscoring their ability to mediate distal host–microbiota communication and directly affect tumor immunogenicity [89]. Similarly, bEVs derived from *Bacillus licheniformis* reduce LC cell viability and proliferation; when combined with doxorubicin (DOX), they enhance apoptosis through the upregulation of p53, p21, caspase-3/9, and Bax while suppressing Bcl-2 [115].

The engineering of bEV membranes to display tumor-specific antigens represents a promising strategy for personalized immunotherapy. Modular platforms allow decoration of bEVs with multiple tumor-associated epitopes, facilitating targeted immune activation. Although currently supported by preclinical murine models, these approaches pave the way for personalized nanovaccine carriers and enhanced drug delivery systems [116].

Gram-negative bEVs are particularly attractive as drug carriers due to their stability and permeability to genetic and chemical modification. For instance, doxorubicin-loaded bEVs from attenuated *Klebsiella pneumoniae* efficiently target NSCLC cells and induce potent cytotoxicity in vitro [113,117].

Beyond therapeutic applications, bEVs serve as valuable non-invasive biomarkers. bEVs isolated from bronchoalveolar lavage fluid or peripheral blood enable the detection of LC-specific proteins and miRNAs, supporting early diagnosis, prognosis, and real-time monitoring of treatment responses [118].

Engineered bEVs also act as RNA delivery vehicles; encapsulation of siRNAs targeting oncogenic KRAS mutations has suppressed tumor growth in pancreatic cancer models, suggesting translational potential for KRAS-driven LC [14,119].

### Clinical Trials Investigating Microbiota-Based Therapies in NSCLC

Over the past decade, evidence has underscored the critical role of both the gut and lung microbiota in modulating systemic immunity and responses to cancer immunotherapies [120]. While correlations between gut dysbiosis and resistance to ICIs in LC are well documented, direct causal evidence remains limited. Preclinical models using fecal microbiota transplantation (FMT) from ICI responders or non-responders into germ-free or antibiotic-treated mice support a causal role for microbiota in modulating antitumor immunity. Building on these findings, early-phase clinical trials are exploring FMT, probiotics, and other microbiota-modulating interventions to enhance ICI efficacy in NSCLC [121].

The rationale for these strategies is based on the microbiome’s capacity to enhance tumor immunogenicity, promote antigen presentation, and counteract immunotherapy resistance [122].

Microbial components influence both systemic and tumor-level immune landscapes, offering avenues to boost ICI responses [123]. However, current trials show several challenges: They generally enroll small patient cohorts, which limits statistical power and generalizability; they are mostly early-phase studies focused on safety rather than survival outcomes; and patient heterogeneity in terms of prior therapies, ICI exposure, and disease stage complicates the interpretation of results. Follow-up periods are often short or undefined, restricting the evaluation of long-term endpoints such as progression-free or overall survival. Moreover, mechanistic analyses are inconsistently performed. Longitudinal microbiome profiling is lacking, and validated microbiome-based biomarkers to stratify patients are absent. Safety concerns also remain, particularly with FMT or live biotherapeutics in immunocompromised patients. Despite these limitations, ongoing research and technological advances in bEV engineering, microbiota modulation, and targeted delivery systems underscore their translational potential as both therapeutic and diagnostic tools in LC management. Table 2 provides a summary of selected ongoing or recently completed clinical trials focused on microbiota-based therapies in NSCLC (source: https://clinicaltrials.gov, accessed on 7 October 2025).

## 6. Limitations and Challenges

**Figure 3:**
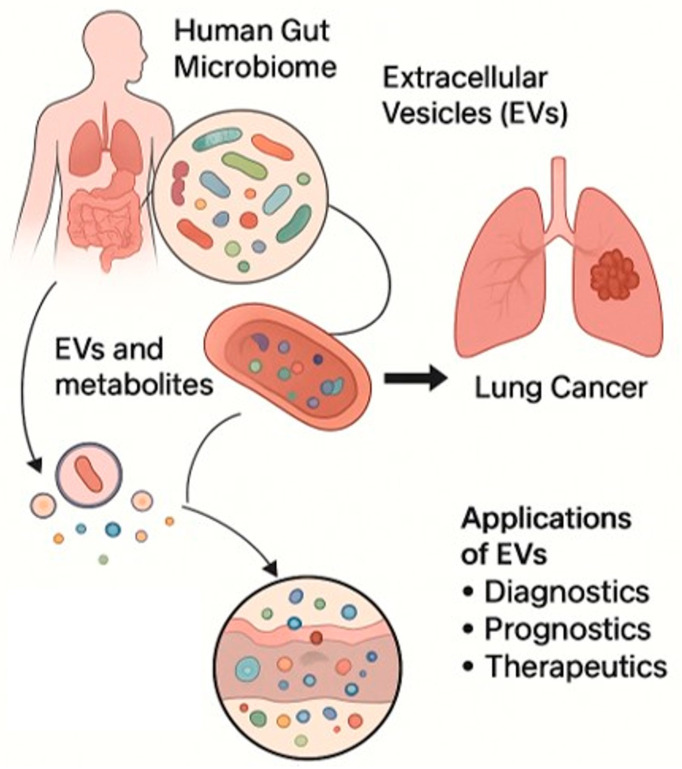
Schematic representation of bEVs released by the human gut microbiome that can influence the lung cancer TME and potential applications of EVs as diagnostic, prognostic, and therapeutic tools. Microsoft Copilot was used for the graphical rendering of the figure.

Despite the growing recognition of the biomedical potential of bEVs in LC, several technical and biological challenges currently limit their full translational application. A primary issue is the difficulty in accurately distinguishing host-derived EVs from bacterial EVs in complex biological fluids such as blood and bronchoalveolar lavage [124]. Current isolation methods often yield heterogeneous EV populations, reducing the specificity and reproducibility of molecular analyses. Standardized protocols for bEV isolation, purification, and characterization are lacking [62], and variability in vesicle yield, purity, and functional assessments across laboratories hampers comparability and robust validation. High interindividual variability within the microbiota–immune–tumor axis further complicates the translation of preclinical findings []. Factors such as host genetics, diet, medication use, and environmental exposures alter and shape the microbiota composition, consequently influencing bEV production and function. Moreover, the mechanisms governing bEV biogenesis, bEV selection, organ-specific tropism, and immunomodulatory capacity remain poorly defined. Further studies are needed to elucidated how microbial and host factors dictate these processes and to identify specific bEV cargos with diagnostic or therapeutic relevance (Figure 3) [14].

Incorporating 3D organoid modeling will further enhance predictive accuracy and accelerate translational progress. Addressing these challenges through methodological standardization, comprehensive mechanistic studies, and large-scale clinical validation will be essential to unlock the full potential of bEVs in LC diagnostics, therapy, and immunomodulation [125].

Clinical and translational studies evaluating bEVs as diagnostic biomarkers for LC remain limited. While some bEV-derived indices show promising sensitivity, specificity and predictive values are often suboptimal. For instance, a serum bEV-based “BTS index” in renal cell carcinoma achieved 89–91% sensitivity but only 38–40% specificity [126]. In gastric cancer, serum or urine bEVs improved detection compared with conventional tumor markers, particularly in early-stage disease, though precise positive and negative predictive values remain largely undefined. A urine-derived bEV logistic regression model reported an AUC of 0.823, a sensitivity score of 67.7%, a specificity value of 84.9%, and an overall accuracy score of 76.1% [127]. More recently, a serum bEV-based BAF index demonstrated high sensitivity in both discovery and validation cohorts, although positive and negative predictive values are not yet fully established [128].

## 7. Conclusions and New Perspectives

Recent advances have highlighted the integral role of the gut–lung microbiome axis, bEVs, and miRNAs in regulating critical signaling pathways implicated in LC development and responses to immune checkpoint blockade. Among these, circulating bEVs present in various body fluids have emerged as rich reservoirs of biological information, as they are capable of reflecting both normal physiological states and disease-associated alterations.

**Figure 4:**
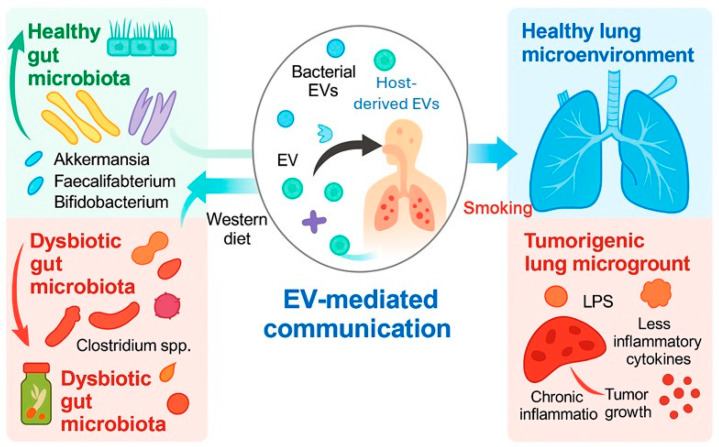
Schematic diagram of bEVs as molecular bridges in the gut–lung axis. Environmental and microbial factors determine the bEV cargo, influencing immune regulation, inflammation, and tumor progression. Microsoft Copilot was used for the graphical rendering of the figure.

As key mediators of gut–lung communication, bEVs facilitate inter-organ signaling, modulate immune responses, and contribute to the remodeling of the TME. Increasing evidence suggests that environmental factors and the microbiota composition critically influence the molecular cargo and functional characteristics of bEVs, thereby affecting tumor progression and therapeutic outcomes (Figure 4). Among bEV cargos, microbial nucleic acids hold the greatest promise for clinical translation. In particular, bacterial DNA fragments, including 16S-derived signatures, currently represent the most clinically supported bEV-associated candidates for early LC detection. While LPS signatures and bacterial proteins are biologically plausible and supported by preclinical studies, they remain to be directly validated in clinical LC cohorts.

## Figures and Tables

**Table 1:** Summary of bacteria and their EV cargo involved in cancers. Abbreviations: NSCLC: Non-Small Cell Lung Cancer, GC: Gastric Cancer, CRC: Colon–Rectal Cancer, LPS: lipopolysaccharide, PD 1:Programmed Cell Death Protein 1, PD L1: Programmed Death-Ligand 1, TLR: Toll-Like Receptor, PTEN: Phosphatase And Tensin Homolog, NF-κB: Nuclear Factor Kappa-Light-Chain-Enhancer of Activated B Cells, PI3K: Phosphatidylinositol 3-Kinase, AKT: Protein Kinase B.

Bacterial Species	bEV Cargo	Experimental Model	Mechanism/Pathway Activated	Effect on Lung Cancer	Ref(s)
*Pseudomonas aeruginosa*	LPS miR-21 miR-155	Lung epithelial cells	TLR2/4 → NF-κB, PI3K/AKT	Inflammation immune evasion chemoresistance	[93]
*Helicobacter pylori*	miR-155	GC	Downregulation of Bax	Apoptosis resistance	[79]
*Bifidobacterium* spp.	PD-L1-inducing components	NSCLC	TLR4 → NF-κB → PD-L1 upregulation	Modulation of response to anti-PD-1 therapy	[26]
*Akkermansia muciniphila*	PD-1	NSCLC		PD-1 blockade	[87]
*Fusobacterium nucleatum*	miR-21	CRC	PTEN suppression → cell proliferation	Tumor growth promotion	[65]
*Klebsiella pneumoniae* (attenuated)	DOX-loaded bEVs	NSCLC	Intracellular drug delivery	Cytotoxicity in NSCLC cells	[113]
*Bacillus licheniformis*	Native bEV proteins	liver, stomach pancreas cancer	↑ p53, ↑ p21, ↑ caspase-3/9, ↓ Bcl-2	Induction of apoptosis, inhibition of proliferation	[94]

**Table 2:** Summary of selected clinical trials focused on microbiota-based therapies in NSCLC. Abbreviations: NSCLC: Non-Small Cell Lung Cancer, FMT: Fecal Microbiota Transplantation, ICI: Immune Checkpoint Inhibitor, PD-1: Programmed Cell Death Protein 1.

Trial ID	Intervention/Therapy	Key Outcomes
NCT04951583	FMT + ICI	Patients with metastatic NSCLC (and melanoma) receiving ICI + FMT. Objective: evaluate the combined antitumor activity
NCT05008861	Encapsulated FMT combined with anti-PD-1/PD-L1 therapy	Patients with locally advanced or metastatic NSCLC after first-line PD-1/PD-L1 therapy. Assessing safety and impact on the gut microbiota composition
NCT04699721	Neoadjuvant chemo-immunotherapy + probiotics in resectable NSCLC	Evaluates safety and efficacy of probiotic supplementation in patients undergoing neoadjuvant chemo-immunotherapy for resectable NSCLC.

## Data Availability

Not applicable.
